# Chimeric Antigens Receptor T Cell Therapy Improve the Prognosis of Pediatric Acute Lymphoblastic Leukemia With Persistent/Recurrent Minimal Residual Disease in First Complete Remission

**DOI:** 10.3389/fimmu.2021.731435

**Published:** 2022-01-07

**Authors:** Guan-hua Hu, Yi-fei Cheng, Ying-xi Zuo, Ying-jun Chang, Pan Suo, Jun Wu, Yue-ping Jia, Ai-dong Lu, Ying-chun Li, Yu Wang, Shun-chang Jiao, Long-ji Zhang, Xiang-yu Zhao, Chen-hua Yan, Lan-ping Xu, Xiao-hui Zhang, Kai-yan Liu, Yu Wang, Le-ping Zhang, Xiao-jun Huang

**Affiliations:** ^1^ Peking University People’s Hospital, Peking University Institute of Hematology, National Clinical Research Center for Hematologic Disease, Beijing Key Laboratory of Hematopoietic Stem Cell Transplantation, Peking-Tsinghua Center for Life Science, Research Unit of Key Technique for Diagnosis and Treatment of Hematologic Malignancies, Chinese Academic of Medical Sciences, Beijing, China; ^2^ Department of Pediatrics, Peking University People’s Hospital, Peking University, Beijing, China; ^3^ Beijing Yongtai Reike Biotechnology Company Ltd, Beijing, China; ^4^ Chinese People Liberation Army (PLA) General Hospital, Beijing, China; ^5^ Shenzhen Geno-immune Medical Institute, Shenzhen, China

**Keywords:** measurable residual disease, chimeric antigen receptor T-cell, pre-empty therapy, acute lymphocyte leukemia, pediatrics

## Abstract

**Background:**

The presence of minimal residual disease (MRD) is an independent risk factor for poor prognosis in patients with acute lymphoblastic leukemia (ALL). Moreover, the role of chimeric antigen receptor T-cell (CAR-T) therapy in patients with MRD is currently unclear.

**Methods:**

We conducted a prospective study to investigate the role of CAR-T therapy in patients with persistent/recurrent MRD-positive ALL in first remission.

**Results:**

A total of 77 patients who had persistent/recurrent MRD were included. Of these patients, 43 were enrolled in the CAR-T group, 20 received chemotherapy as a bridge to allogeneic hematopoietic cell transplantation (allo-HSCT), and 14 patients received intensified chemotherapy. MRD negativity was achieved in 90.7% of the patients after CAR-T infusion. Patients who received CAR-T therapy had a higher 3-year leukemia-free survival (LFS) than patients who did not (77.8% *vs.* 51.1%, P = 0.033). Furthermore, patients in the CAR-T group had a higher 3-year LFS than those in the chemotherapy bridge-to-allo-HSCT group [77.8% (95% CI, 64.8–90.7%) *vs.* 68.7% (95% CI, 47.7–89.6%), P = 0.575] and had a significantly higher 3-year LFS than those in the intensified chemotherapy group [77.8% (95% CI, 64.8–90.7%) *vs.* 28.6% (95% CI, 4.9–52.3%), P = 0.001]. Among the patients who received CAR-T therapy, eight were not bridged to allo-HSCT, and six (75%) remained in remission with a median follow-up of 23.0 months after CAR-T infusion.

**Conclusions:**

Our findings show that CAR-T therapy can effectively eliminate MRD and improve survival in patients with a suboptimal MRD response.

## Background

Acute lymphoblastic leukemia (ALL) is the most common pediatric cancer. Despite remarkable improvements in the prognosis of ALL over the past decades, treating relapsed ALL remains challenging. Many studies have shown that eliminating minimal residual disease (MRD), which is one of the most important criteria for risk stratification, reduces relapse (1, 2). The US Food and Drug Administration claimed that the persistence of MRD is associated with poor prognosis, regardless of trial approach and detection methods, and highlighted the need for interventions for patients in first complete remission (CR1) (3). A previous study in our institute also showed that persistent/recurrent MRD was the most significant adverse prognostic indicator in pediatric ALL (4). Thus, eradicating MRD in CR1 is essential.

Allogeneic hematopoietic stem cell transplantation (allo-HSCT) is recommended as a frontline treatment for high-risk and relapsed ALL. Several studies have suggested that allo-HSCT is associated with lower relapse risk than chemotherapy in patients with MRD in CR1 (5, 6). However, Zhao et al. indicated that the 3-year cumulative incidence of relapse was significantly lower in patients with pre-HSCT MRD negativity than in those with pre-HSCT MRD positivity (16% *vs.* 31%, P < 0.001). St. Jude Children’s Research Hospital also reported that persistent MRD at the time of HSCT was associated with high relapse rates and transplant-related mortality (TRM) (7). HSCT and intensification of chemotherapy to decrease pre-HSCT MRD carry a substantial risk of morbidity and mortality in the presence of MRD. Therefore, novel therapeutic approaches that can effectively eliminate MRD are urgently needed.

Recently, chimeric antigen receptor T-cell (CAR-T) therapy has been reported to be the most promising approach for the treatment of refractory/relapsed (R/R) B-cell ALL (8). In the ELIANA trial, which included children and young adults with R/R B-ALL, the response rate was 81%, and the leukemia-free survival (LFS) among responders was 62% at 24 months (9). Several groups have shown that most of patients become MRD-negative and maintain their status for several months after CAR-T infusion. Moreover, a previous study in our institute showed that CAR-T infusion was effective in patients with MRD and with no responsive to donor lymphocytes infusion after allo-HSCT (10), suggesting that CAR-T therapy has the potential to induce deeper remission while reducing toxicity. However, the short-term and long-term effects of CAR-T on patients with MRD have not been assessed. Several issues that need to be explored include whether CAR-T therapy can efficiently eradicate MRD and reach a satisfactory response rate similar to those of previous reports on R/R ALL, whether CAR-T therapy can improve the long-term survival of patients with MRD, and whether sustained remission can be achieved with the application of CAR-T therapy without allo-HSCT. To the best of our knowledge, this study is the first prospective study to explore the role of CAR-T therapy in patients with persistent/recurrent MRD in CR1.

## Methods

### Patients

**Figure 1:**
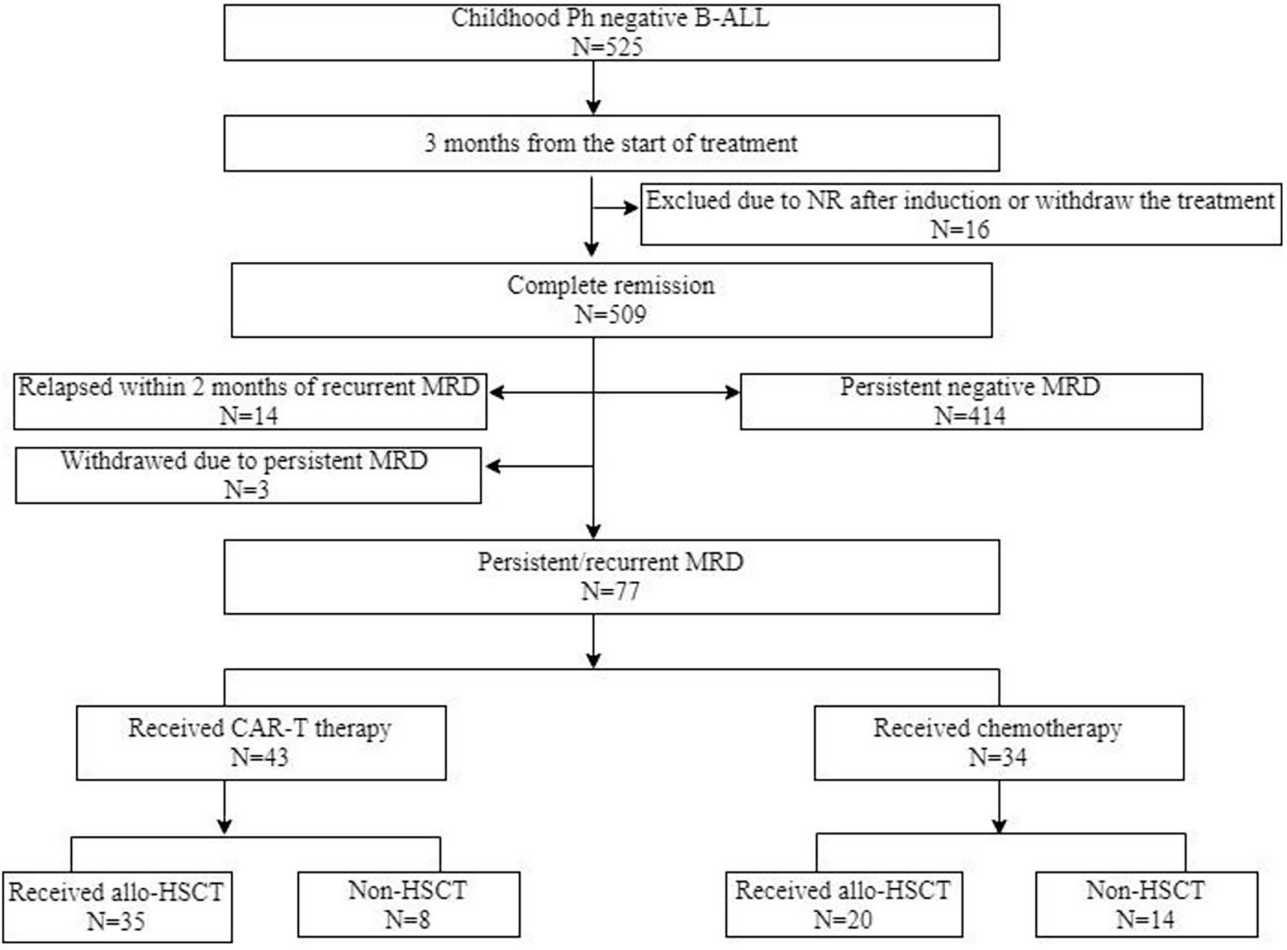
Diagram of patients enrolled in this study. CAR-T, chimeric antigen receptor T cells; ALL, acute lymphoblastic leukemia; allo-HSCT, allogeneric haematopoietic stem cell transplantation; MRD, measurable residual disease; NR, non-remission; Ph, Philadelphia chromosome.

In this single-center, prospective study, a total of 525 patients who were newly diagnosed with Philadelphia chromosome-negative B-ALL between January 2015 and September 2019 were included (**Figure 1**). The inclusion criteria were (1) age of 1–18 years (2), achieved complete remission (CR) after induction chemotherapy (3), persistent positive MRD within three months from the start of treatment, and (4) achieved MRD negativity and the conversion of negative to positive MRD during consolidation chemotherapy. The exclusion criteria included (1) morphological relapse within two months of recurrent MRD and (2) severe heart, kidney, or liver disease. This study was approved by the Peking University People’s Hospital review board. All patients’ legal guardians provided written informed consent documents in accordance with the Declaration of Helsinki.

### CAR-T Protocols

The lymphodepleting chemotherapy before CAR-T therapy included fludarabine (25 mg/m^2^/d on days –5 to –3) and cyclophosphamide (250 mg/m2/d on days –5 to –3). Multiple up-to-date CAR-T engineering technologies have been summarized (11). Anti-CD19 CAR-T cells constructed with a 4-1BB (79%) or CD28 (21%) costimulatory domain were generated *via* lentiviral vector from fresh leukapheresis material in this study.

For the CAR-T with a 4-1BB costimulatory domain, peripheral blood mononuclear cells (PBMCs) were collected from patients and stimulated with dynabeads coated with anti-CD3 and anti-CD28 mAbs (Thermo Fisher Scientific). Activated T cells were transduced with lentiviral vector encoding the anti-CD19 CAR construct consisting of CD19 recognition domain, transmembrane link domain, 4-1BB intracellular domain, CD3ζ intracellular domain. After lentiviral transduction, the 4-1BB CAR-T-19 cells were cultured in medium supplemented with 500 IU/ml IL-2 at 37°C/5% CO2 for approximately 5 to 11 days to obtain sufficient cells for infusion.

For the CAR-T with a CD28 costimulatory domain, peripheral blood mononuclear cells (PBMCs) were acquired through apheresis from the patients at relapses with sufficient lymphocyte counts, and the T cells were selected using CD3 magnetic beads. CD28 monoclonal antibodies were added for T cell activation *in vitro*. The activated T cells were transduced with the 4SCAR19 lentiviral vector encoding the CD19 CAR carrying a “safety switch”—iCasp9 for 3-5 days. The CAR-T cells were cultured in AIM-V (Invitrogen, San Diego, CA, USA) medium supplemented with IL-2, IL-7, and IL-15 at 37°C/5% CO2 for approximately 5–7 days to obtain sufficient cells for infusion.

Real-time quantitative polymerase chain reaction was used to quantify the level of the CAR gene (<100 copies/μg DNA was defined as negative). Flow cytometry (FCM) was performed to determine the transduction efficiency and the ratio of B cells in peripheral blood and bone marrow after CAR-T infusion. For patients who chose to be enrolled in the observation group after CAR-T infusion, the levels of the CAR gene and B cells were assessed every month, and maintenance chemotherapy was administered if CAR T cells were not detected *in vivo* and/or B cells were recovered.

### Transplant Protocols

The conditioning regimen for allo-HSCT was in accordance with previous reports (12, 13). Patients who received an HLA-mismatched HSCT received a regimen that included cytarabine (4 g/m2/day IV, days −10 and −9), busulfan (3.2 mg/kg/day IV, days −8 to −6), cyclophosphamide (1.8 g/m2/day IV, days −5 and −4), semustine (250 mg/m2 PO, day −3), and antithymocyte globulin (ATG) (2.5 mg/kg/day IV, days −5 to −2). Patients who received an HLA-identical HSCT were treated with a regimen identical to that of the patients who received an HLA-mismatched HSCT, but without ATG. All patients received acute graft-versus-host disease (aGVHD) prophylaxis consisting of cyclosporine A, mycophenolate mofetil, and a short-term methotrexate regimen.

### Chemotherapy Protocols

The intensified chemotherapy regimens were in accordance with a previous report (14). These regimens included (1) an induction therapy (2), a consolidation therapy with two cycles of a re-induction block in between, and (3) a maintenance therapy. The induction and re-induction chemotherapy regimens consisted of vincristine, idarubicin, cyclophosphamide, and L-asparaginase. The consolidation chemotherapy regimens were comprised of methotrexate (MTX), vincristine, and peg-aspargase; and cytarabine (Ara-c), idarubicin, fosfamide, etoposide, and vincristine. The maintenance chemotherapy regimens included mercaptopurine (50 mg/m2/d PO, daily) and methotrexate (20 mg/m2/d IM, weekly). Patients in the chemotherapy bridge-to-allo-HSCT group received MTX (2–3 g/m2/d IV) and/or Ara-c (2 g/m2/d IV, days 1 to 3) based chemotherapy regimens.

### Detection of MRD

A panel of eight antibody combinations, which included cCD3, mCD3, CD2, CD5, CD7, CD10, CD19, CD20, CD34, CD38, CD45, CD58, CD99, CD123, and cTDT, were used for MRD detection. The standardized assays and quality controls were consistent with those of previous reports (15). Any MRD level was considered positive. MRD was assessed every month until MRD negativity, every two to three months during consolidation chemotherapy, and every six months during maintenance chemotherapy for patients in the chemotherapy group. MRD was assessed every month until one year of CAR-T therapy and every two to three months until two years of CAR-T therapy for patients in the CAR-T group. MRD was assessed at 1, 2, 3, 4.5, 6, 9, and 12 months post-HSCT and at six-month intervals thereafter for patients in the HSCT group.

### Definitions

CR was defined as the presence of <5% blasts in the bone marrow, an absolute neutrophil count of >1 × 10^9^/L, a platelet count of >100 × 109/L, and the absence of extramedullary disease. Recurrence of ≥5% bone marrow blasts and/or the development of extramedullary disease were defined as a relapse. Recurrent MRD was defined as two MRD-positive samples at an interval of one month in a patient who was previously MRD-negative. Non-relapse-related mortality (NRM), aGVHD, and chronic GVHD (cGVHD) were defined as previously described (12). Cytokine release syndrome (CRS) grading was based on the National Cancer Institute (NCI) consensus CRS scoring system.

### End Points and Statistical Analysis

The primary endpoint is LFS. The secondary endpoints are MRD negativity rate, overall survival (OS), and safety. LFS was measured from the time when CR was achieved; events of LFS included death in CR1 or relapse. MRD negativity was associated with an undetectable MRD by FCM. The event of OS was death at the date of the last follow-up. The patients’ characteristics were compared using the chi-square test or Fisher’s exact test for categorical variables and the Mann–Whitney rank test or Student’s t-test for continuous variables. The Kaplan-Meier method was used to analyze the LFS and OS. Comparisons between different LFS and OS probabilities were performed using the log-rank test. Multivariate analysis was performed using the Cox proportional hazards regression model. Statistical significance was set at P < 0.05. SPSS version 23.0 (SPSS Inc., Chicago, IL, USA) and R version 3.5.3 (R Foundation for Statistical Computing, Vienna, Austria) were used for data analysis.

## Results

### Patients’ Characteristics

A total of 525 patients diagnosed with Philadelphia chromosome-negative B-ALL were included in this study; 448 patients were excluded due to persistent negative MRD (n = 414), relapse within two months of recurrent MRD (n = 14), withdrawal due to personal reasons (n = 12), failure to achieve CR after induction chemotherapy (n = 6), and loss to follow-up (n = 2). Of the 77 patients with persistent/recurrent MRD who were screened and encouraged to receive CAR-T therapy, 43 patients were enrolled in the CAR-T group. The remaining patients were divided into the chemotherapy bridge-to-allo-HSCT group (n = 20) and the intensified chemotherapy group (n = 14) according to their personal willingness, economic background, and donor availability. After a month of CAR-T infusion, patients who failed to achieve MRD negativity received allo-HSCT (n = 4), and patients who achieved MRD negativity were divided into the bridge-to-allo-HSCT group (n = 31) and the observation group (n = 8). **Tables 1** and **2** show the characteristics of the patients, and the baseline characteristics of the patients who received CAR-T therapy and those who did not were comparable.

**Table 1:** Characteristics of patients stratified by CAR-T and non-CAR-T group.

Characteristics	CAR-T Group	Non-CAR-T Group	P value
Number of patients	43	34	
Median age (range), years	8.3 (1–17)	8.2 (1–17)	0.978
Male sex, n (%)	23 (53.5)	22 (64.7)	0.359
Cytogenetic risk group			
Low-risk, n (%)	29 (67.4)	19 (55.8)	0.348
High-risk, n (%)	14 (32.6)	15 (44.2)	0.348
Fusion genes, n (%)			
*MLL-AF4*	2 (4.6)	4 (11.7)	0.251
*E2A-PBX1*	2 (4.6)	2 (5.8)	0.810
*TEL-AML1*	6 (13.9)	2(5.8)	0.252
*E2A-HLF*	1 (2.3)	1 (2.9)	0.867
High hyperdiploid	6 (13.9)	3 (8.8)	0.489
High risk gene mutation, n (%)	14 (32.5)	13 (38.2)	0.604
*IKZF1*	6 (13.9)	8 (23.4)	
*JAK2*	3 (6.9)	4 (11.7)	
*CRLF2*	3(6.9)	1 (2.9)	
*PDGFRB*	2 (4.6)	0 (0.0)	
Extramedullary infiltration, n (%)	5 (11.6)	4 (11.7)	0.628
Persistent positive MRD, n (%)	17 (39.5)	16 (47.0)	0.259
Recurrent positive MRD, n (%)	26 (60.5)	18 (53.0)	0.488
MRD > 0.1% at any checking points, n (%)	29 (67.4)	27 (79.4)	0.307
MRD > 1% at any checking points, n (%)	15 (34.8)	9 (26.4)	0.467
Levels of recurrent MRD (%)	0.52 (0.01-5.09)	0.58 (0.004-3.3)	0.843
Follow-up time (range), months	37.4 (7.0-70.0)	45.0 (7.0-70.0)	0.518

CAR-T, chimeric antigen receptor T cells; MRD, minimal residual disease.

**Table 2:** Characteristics of patients stratified by CAR-T bridge to allo-HSCT and chemotherapy bridge to allo-HSCT.

Characteristics	CAR-T Bridge to Allo-HSCT	Chemotherapy Bridge to Allo-HSCT	P value
Number of patients	35	20	
Median age (range), years	8.4 (1–17)	9.3 (1–17)	0.546
Male sex, n (%)	17(48.6)	11 (55.0)	0.781
Median level of pre-HSCT MRD (range), %	0.04 (0.00-0.77)	0.06 (0.00-1.5)	0.754
Median time from diagnosis to allo-HSCT (range), months	8.2 (3–14)	7.5 (4–20)	0.348
Donor-recipient sex match grafts, n (%)			
Male-male	17 (48.5)	12 (60)	0.575
Male-female	11 (31.4)	7 (35.0)	0.786
Female-male	3 (8.5)	0 (0.0)	0.182
Female-female	4 (11.4)	1 (5.0)	0.429
Donor-recipient relationship, n (%)			
Father-child	26 (74.2)	18 (90.0)	0.165
Mother-child	3 (8.6)	1 (5.0)	0.627
Brother-brother	3 (8.6)	0(0.0)	0.182
Sister-brother	3 (8.6)	1 (5.0)	0.627
ABO matched grafts, n (%)			
Matched	17 (48.6)	12 (60.0)	0.575
Major mismatch	5 (14.3)	3 (15.0)	0.943
Minor mismatch	11 (31.4)	4 (20.0)	0.364
Bidirectional mismatch	2 (5.7)	1 (5.0)	0.911
Cell compositions in grafts, mean (range)			
Infused nuclear cells, 10^8^/kg, mean (range)	9.5 (7.0-14.0)	9.5 (6.6-13.91)	0.792
infused CD34^+^cells, 10^6^/kg, mean (range)	3.4 (1.2-9.8)	2.8 (0.9-4.7)	0.543
Median time of neutrophil engraftment (range), days	13.1 (10–18)	13.9 (10–19)	0.965
Median time of platelet engraftment (range), days	15.6 (9–46)	20.3 (10–43)	0.687
Grade II-IV aGVHD, %	22	21	0.758
Chronic GVHD, %	43	40	0.810
Moderate to severe cGVHD, %	19	21	0.897
3-years probability of LFS, %	75.0	68.7	0.586
3-years probability of OS, %	85.6	73.3	0.920

CAR-T, chimeric antigen receptor T cells; GVHD, graft-versus-host disease; HSCT, allogeneic hematopoietic stem cell transplantation; LFS, leukemia-free survival; MRD, minimal residual disease; OS, overall survival.

### CAR-T Therapy

In this study, 43 patients received CAR-T therapy. The median dose of infused CAR T cells was 3.85 × 10^6^ (0.45–8.45 × 10^6^)/kg. The viability of CAR-T pre-infusion was 93.4% (75.0-99.1%), transduction efficiency of CAR-T pre-infusion was 29.5% (8.7-83.8%). CAR T cells rapidly expanded during the first month in 42 (97.6%) patients. The mean CAR-T count at peak expansion was 303,238 (1,250–1,890,000), and the median time to peak expansion was 11.6 (7–17) days. One patient had unsatisfactory peak CAR-T counts (1,250 copies at peak expansion).

The median MRD level pre-lymphodepletion was 0.22% (0.01–2.86%), and the rate of patients who achieved MRD negativity after a month of CAR-T infusion was 90.7%. Patients who achieved MRD negativity and those who did not received comparable doses of CAR T cells (3.9 × 10^6^/kg *vs.* 5.0 × 10^6^/kg, P = 0.65), but the peak CAR-T counts of patients who achieved MRD negativity were significantly higher than those of patients who failed to achieve MRD negativity (362,350 copies *vs.* 70,323 copies, P = 0.02). Among the patients who failed to achieve MRD negativity after a month of CAR-T infusion (n = 4), one had unsatisfactory peak CAR-T counts, and another had undetectable CAR-T counts at 1 month after CAR T-cell infusion.

Among the patients who received CAR-T therapy, 23 (53.8%) had CRS of any grade, and six (13.9%) had severe CRS (grades 3 and 4). Neurological adverse events occurred in six (13.9%) patients, of which four experienced headache and confusion, one had seizures, and another had encephalopathy. No CAR-T-related mortality was observed.

All patients who failed to achieve MRD negativity after CAR-T therapy received allo-HSCT. Among the patients who achieved MRD negativity after CAR-T infusion (n = 39), 31 were bridged to allo-HSCT after CAR-T therapy, while eight were not (**Table 3**). Among patients who bridged to allo-HSCT after CAR-T therapy, 26 of them received 4-1 BB CAR-T, CAR-T cell can be detected before allo-HSCT in 24 patients who received 4-1 BB CAR-T. Among patients who bridged to allo-HSCT after CAR-T therapy, 9 of them received CD28 CAR-T (**Table 4**), the level of CAR-T cell after infusion was not monitored.

**Table 3:** Clinical features and outcomes of patients who did not bridge to allo-HSCT after CAR-T therapy.

Patient	Cyto/mol abn	MRD Before Lymphodepletion (%)	Total CAR-T cells/kg infused	Time of Persistence of CAR-T Cell (months)	Time of B Cell Recovery (months)	Treatment After CAR-T Disappeared	Outcome After CAR-T
1	No	0.08	3.4×10^6^	4	2.5	6-MP; MTX	CCR for 29 months
2	hypodiploid	0.02	5.0×10^6^	2.5	1.5	6-MP; MTX	CD19+relapse
3	No	0.03	3.0×10^6^	2.5	6	6-MP; MTX	CCR for 21 months
4	TEL/AML1	0.3	5.3×10^6^	4	4	6-MP; MTX	CCR for 12 months
5	hyperdiploid	0.98	0.45×10^6^	12	7.5	6-MP; MTX	CCR for 14 months
6	IKZF1	0.56	5.0×10^6^	8	without B cell recovery	6-MP; MTX	CCR for 23 months
7	TEL/AML1	0.06	4.6×10^6^	2	2	CD22-CAR-T	CCR for 14 months
8	IKZF1; complex chromosome	0.28	5.0×10^6^	5	without B cell recovery	Chinese medicine	CD19-relapse

CAR-T, chimeric antigen receptor T cells; Cyto/mol abn, cytogenetic/molecular abnormalities; CCR, continuous complete remission; 6-MP, mercaptopurine; MRD, minimal residual disease; MTX, methotrexate.

**Table 4:** Characteristics of patients stratified by 4-1 BB CAR-T and CD28 CAR-T.

Characteristics	4-1BB CAR-T	CD28 CAR-T	P value
Number of patients	34	9	
Median age (range), years	8.0 (2–16)	9.0 (1–17)	0.613
Male sex, n (%)	19 (55.8)	2 (22.2)	0.076
Cytogenetic risk group			
Low-risk, n (%)	23 (67.6)	6 (66.7)	
High-risk, n (%)	11 (32.4)	3 (33.3)	0.956
MRD negativity after one month of CAR-T infusion, n (%)	33 (97.0)	6 (66.7)	0.006
Bridge to allo-HSCT, n (%)	26 (76.5)	9 (100)	0.111
3-years probability of LFS, %	80.7	66.7	0.426
3-years probability of OS, %	91.1	66.7	0.138
CRS of any grade, n (%)	21 (61.7)	2 (22.2)	0.037
Severe CRS (grade 3 and 4), n(%)	6 (17.6)	0 (0)	0.179

CAR-T, chimeric antigen receptor T cells; CRS, cytokine release syndrome; HSCT, allogeneic hematopoietic stem cell transplantation; LFS, leukemia-free survival; MRD, minimal residual disease; OS, overall survival.

In patients who were not bridged to allo-HSCT after CAR-T therapy, the median dose of infused CAR T cells was 3.96 × 10^6^ (0.45–5.3 × 10^6^)/kg, the median persistence time of CAR T cells was 5.0 (2–12) months, and the median recovery time of B cells was 3.9 (1.5–7.5) months. B cells were not recovered in two patients until the last follow-up. One of them remained in CR after 23 months of CAR-T infusion, while the other patient with CD19 negativity relapsed.

### Allo-HSCT

In this study, 55 patients received allo-HSCT. A total of 35 patients received CAR-T therapy before allo-HSCT, and the median time from CAR-T therapy to allo-HSCT was 67 days. The other 20 patients received chemotherapy before allo-HSCT. Thirty (85.7%) patients in the CAR-T bridge-to-allo-HSCT group were MRD-negative pre-HSCT, while the other five patients were MRD-positive with a median MRD level of 0.17% (0.01–0.77%). Fifteen (75%) patients in the chemotherapy bridge-to-allo-HSCT group were MRD-negative pre-HSCT, while the other five patients were MRD-positive with a median MRD level of 0.44% (0.01–1.5%).

Of the patients who received allo-HSCT, five received allo-HSCT from matched sibling donors, while the others received haplo-HSCT. Patients achieved neutrophil engraftment at a median time of 13 (10–35) days, and all patients achieved platelet engraftment at a median time of 14 (7–58) days. The cumulative 100-day incidence of aGVHD grades II–IV and grades III–IV in the CAR-T bridge-to-allo-HSCT group were similar to those of the chemotherapy bridge-to-allo-HSCT group [24% (95% CI, 17–27%) *vs.* 23% (95% CI, 12–32%), P = 0.956; 8% (95% CI, 4–12%) *vs.* 6% (95% CI, 3–11%), P = 0.818]. The cumulative 3-year incidence of total cGVHD and severe cGVHD in the CAR-T bridge-to-allo-HSCT group were also similar to those of the chemotherapy bridge-to-allo-HSCT group [56% (95% CI, 38–65%) *vs.* 49% (95% CI, 39–55%), P = 0.687; 12% (95% CI, 6–19%) *vs.* 11% (95% CI, 5–15%), P = 0.918]. The cumulative 3-year incidence of NRM was 3% (95% CI, 1–6%).

### Chemotherapy

Of the patients who received intensified chemotherapy without allo-HSCT, nine (64.2%) achieved MRD negativity. No serious treatment-related toxicity or TRM was observed.

### Relapse, LFS, and OS

Between January 1, 2015 and December 31, 2020, the median follow-up time for surviving patients was 44.0 (18.0–70.0) months. Of patients in the CAR-T group, 10 (23.2%) relapsed (four withdrew, three achieved second CR with allo-HSCT, two achieved second CR with CD22-CAR-T therapy, and one abandoned further treatment after no response to CD22-CAR-T therapy). Relapse occurred at a median time of 9.6 (4–17) months after CAR-T infusion. Nine (20.9%) patients experienced a CD19-positive relapse, while one (2.3%) patient experienced a CD19-negative relapse. Of the patients in the chemotherapy bridge-to-allo-HSCT group, six (30%) relapsed (four withdrew and two achieved second CR with allo-HSCT). Of the patients who received intensified chemotherapy, 10 (71.4%) relapsed (five withdrew, three achieved second CR with allo-HSCT, and two failed to achieve second CR with salvage chemotherapy). At the last follow-up, 17 (22.0%) patients died of relapse, and two (2.5%) patients died of transplant-related complications.

**Figure 2:**
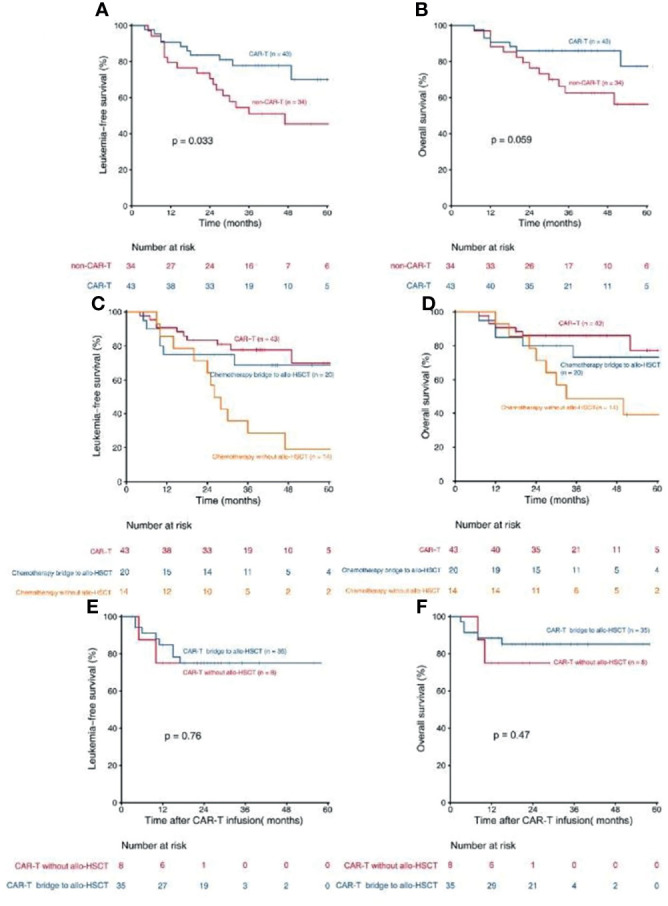
Kaplan- Meier estimates for patients with persistent/recurrent MRD. **(A)** LFS rates for patients received CAR-T therapy and patients did not receive CAR-T theraphy; **(B)** OS rates for patients received CAR-T therapy and patients did not receive CAR-T therapy; **(C)** LFS rates for patients received CAR-T therapy, patients received chemotherapy bridge to allo-HSCT and patients received chemotherapy without allo-HSCT; **(D)** OS rates for patients received CAR-T therapy, patients received chemotherapy bridge to allo-HSCT and patients received chemotherapy without allo-HSCT; **(E)** LFS rates for CAR-T patients who bridge to allo-HSCT and who did not; **(F)** Os rates for CAR-T patients who bridge to allo-HSCT and who did not.

Patients who received CAR-T therapy (n = 43) had a higher 3-year LFS [77.8% (95% CI, 65.6–89.9%) *vs.* 51.1% (95% CI, 33.8–68.3), P = 0.033, **Figure 2A**] and a trend of higher OS [86.0% (95% CI, 93.4–75.6%) *vs.* 62.6% (95% CI, 45.5–76.5%), P = 0.059, **Figure 2B**] than those who did not (n = 34). Patients in the CAR-T group (n = 43) also had a trend of higher 3-year LFS than those in the chemotherapy bridge-to-allo-HSCT group (n = 20) [77.8% (95% CI, 64.8–90.7%) *vs.* 68.7% (95% CI, 47.7–89.6%), P = 0.575] and had a significantly higher 3-year LFS than those in the intensified chemotherapy group (n = 14) [77.8% (95% CI, 64.8–90.7%) *vs.* 28.6% (95% CI, 4.9–52.3%), P = 0.001) (**Figure 2C**). The 3-year OS of patients in the CAR-T group (n = 43) tented to be higher than that in the chemotherapy bridge-to-allo-HSCT group (n = 20) [86.0% (95% CI, 75.6–96.3%) *vs.* 73.3% (95% CI, 52.9–93.6%), P = 0.470] and was significantly higher than that in the intensified chemotherapy group (n = 14) [86.0% (95% CI, 75.6–96.3%) *vs.* 49.0% (95% CI, 22.3–75.6%), P = 0.010] (**Figure 2D**).

Multivariate Cox regression modeling showed that MRD ≥1% at any checking point and non-CAR-T therapy were independent risk factors associated with inferior LFS in all patients (**Table 5**).

**Table 5:** Multivariate analysis of factors associated with survival outcomes.

	LFS		OS	
Variable	HR (95%CI)	P	HR (95%CI)	P
**Overall patients**				
Cytogenetic risk group (high-risk *vs.* non-high-risk)	1.326 (0.753-2.335)	0.328	1.781 (0.894-3.631)	0.112
Level of MRD (≥1% *vs.*<1%)	3.659 (1.642-8.155)	0.002	2.424 (0.947-6.203)	0.065
CAR-T therapy (no *vs.* yes)	2.409 (0.999-5.812)	0.049	2.112 (0.733-6.086)	0.166
HSCT (no *vs.* yes)	2.075 (0.890-4.838)	0.091	2.249 (0.832-6.077)	0.110
**Recurrent MRD group**				
Cytogenetic risk group (high-risk *vs.* non-high-risk)	0.783 (0.160-3.831)	0.763	2.546 (0.613-10.572)	0.198
Median time of MRD recurred (<18 months *vs.* ≥18 months)	1.001 (1.095-1.718)	0.430	2.714 (0.537-8.323)	0.044
Level of recurred MRD (≥1% *vs.*<1%)	1.605 (1.895-2.981)	0.000	0.428 (0.069-2.641)	0.361
CAR-T therapy (no *vs.* yes)	9.456 (2.087-42.848)	0.004	4.736 (0.872-25.718)	0.072
HSCT (no *vs.* yes)	6.642 (1.116-39.519)	0.037	23.503 (2.633-209.791)	0.005
**Persistence positive MRD**				
Cytogenetic risk group (high-risk *vs.* non-high-risk)	1.994 (0.547-7.268)	0.295	2.669 (0.669-10.785)	0.168
Level of MRD (≥1% *vs.*<1%)	2.907 (0.804-10.503)	0.104	1.239 (0.296-5.188)	0.769
CAR-T therapy (no *vs.* yes)	1.674 (0.407-6.892)	0.476	0.671 (0.154-2.934)	0.596
HSCT (no *vs.* yes)	3.160 (0.720-13.808)	0.127	1.899 (0.331-10.902)	0.472
**CAR-T therapy group**				
Cytogenetic risk group (high-risk *vs.* non-high-risk)	1.384 (0.305-6.281)	0.674	12.413 (1.275-120.851)	0.030
Level of MRD (≥1% *vs.*<1%)	2.291 (0.560-9.377)	0.249	1.787 (0.304-10.510)	0.521
MRD after CAR-T (positive *vs.* negative)	1.236 (0.206-7.436)	0.817	3.677 (0.497-27.193)	0.202
Bridge to HSCT (no *vs.* yes)	0.633 (0.069-5.816)	0.686	2.528 (0.189-33.717)	0.483
**Allo-HSCT group**				
Cytogenetic risk group (high-risk *vs.* non-high-risk)	0.647 (0.193-2.164)	0.479	1.649 (0.359-7.570)	0.520
Level of MRD (≥1% *vs.*<1%)	5.848 (1.753-19.514)	0.004	5.054 (1.127-22.669)	0.034
Pre-HSCT MRD (negative *vs.* positive)	0.651 (0.168-2.521)	0.534	0.838 (0.512-4.630)	0.840
CAR-T pre-HSCT (no *vs.* yes)	3.010 (0.860-10.466)	0.083	3.425 (0.732-16.022)	0.118
cGVHD (no *vs.* yes)	6.506 (1.518-27.884)	0.012	1.908 (0.401-9.080)	0.417
**Chemotherapy group**				
Cytogenetic risk group (high-risk *vs.* non-high-risk)	1.290 (0.310-5.366)	0.726	1.870 (0.389-8.992)	0.435
Level of MRD (≥1% *vs.*<1%)	4.014 (0.860-18.673)	0.076	9.881 (0.312-11.355)	0.491
Recurrent MRD (yes *vs.* no)	2.771 (0.616-12.474)	0.184	7.875 (0.820-75.640)	0.074

CAR-T, chimeric antigen receptor T cells; CI, confidence interval; GVHD, graft-versus-host disease; HR, hazard ratio; HSCT, allogeneic hematopoietic stem cell transplantation; LFS, leukemia-free survival; MRD, minimal residual disease; OS, overall survival.

### Subgroup Analysis for Patients Who Received CAR-T Therapy

In patients who received CAR-T therapy (n = 43), the LFS and OS of patients who were bridged to allo-HSCT after CAR-T infusion (n = 35) were comparable with those of patients who were not (n = 8) [75.0% (95% CI, 59.9–90.0%)*vs.* 75.0% (95% CI, 45.0–104.9%), P = 0.765, **Figure 2E**; 85.2% (95% CI, 73.2–97.1%) *vs.* 75.0% (95% CI, 45.0–104.9%), P = 0.470, **Figure 2F**]. MRD ≥1% at any checking point pre-CAR-T therapy (n = 15) tended to lower the LFS of the CAR-T group, but the trend was not statistically significant [65.5% (95% CI, 40.8–90.1%) *vs.* 82.9% (95% CI, 67.6–98.1%), P = 0.236], indicating that the negative impact of high-level MRD can be abrogated by CAR-T therapy to some extent. Multivariate Cox regression modeling showed that high-risk cytogenetics was an independent risk factor associated with inferior OS in patients who received CAR-T therapy (**Table 5**).

### Subgroup Analysis for Patients Who Received Allo-HSCT

In patients who received allo-HSCT (n = 55), the 3-year LFS and OS of patients who received CAR-T therapy pre-HSCT (n = 35) were higher than those of patients who did not (n = 20) [75.0% (95% CI, 59.7–90.2%) *vs.* 68.7% (95% CI, 47.7–89.6%), P = 0.586; 85.6% (95% CI, 69.9–97.3%) *vs.* 73.3% (95% CI, 52.9–93.6%), P = 0.382]. MRD ≥1% at any checking point pre-HSCT (n = 16) significantly lowered the LFS and OS of the allo-HSCT group [46.1% (95% CI, 20.2–71.9%) *vs.* 85.5% (95% CI, 73.7–97.2%), P = 0.006; 61.4% (95% CI, 33.9–88.8%) *vs.* 88.7% (95% CI, 78.3–99.0), P = 0.045]. The 3-year LFS of patients with cGVHD (n = 29) [85.9% (95% CI, 71.0–100.7%)] was higher than that of patients without cGVHD (n = 26) [61.4% (95% CI, 41.9–80.8%)] (P = 0.045). Multivariate Cox regression modeling revealed that MRD ≥1% and not having cGVHD were independent risk factors associated with inferior LFS, and MRD ≥1% was also an independent risk factor associated with inferior OS.

### Subgroup Analysis for Patients Who Received Intensified Chemotherapy

In patients who received intensified chemotherapy (n = 14), the 3-year LFS of patients with recurrent MRD (n = 9) was lower than that of patients with persistent MRD (n = 5) [0.0% *vs.* 40.0% [95% CI, 9.0–69.0%], P = 0.350). MRD ≥1% at any checking point (n = 4) tended to lower the LFS (0.0% *vs.* 40.0% [95% CI, 9.6–70.3], P = 0.125).

## Discussion

Multiple studies have demonstrated the effectiveness of CAR-T therapy in treating R/R B-ALL, with consistently high response rates (83–94.3%) (16–19). However, the patients enrolled in previous trials were those who relapsed and were in morphological non-remission (20–22). In a retrospective study, CAR-T therapy was effective in patients with refractory ALL, of which nine had positive MRD (23). However, the survival analysis of patients with MRD was not performed, and the median follow-up time was only seven months. Thus, whether CAR-T therapy can eliminate MRD and improve outcomes in patients with MRD remains unknown. In this study, with a median follow-up of 44.0 months for all patients and 37.4 months for patients who received CAR-T therapy, we observed that MRD negativity after one month of CAR-T infusion was achieved by 90.7% of patients. This proportion was higher than that in patients who did not receive CAR-T infusion (90.7% *vs.* 70.5%, P = 0.036), proving the effectiveness of CAR-T therapy in eliminating MRD. Patients who received CAR-T therapy had a higher 3-year LFS (77.8% *vs.* 51.1%, P = 0.033) than patients who did not, and only one (2.3%) experienced a CD19-negative relapse. Patients who received CAR-T therapy also tended to be a higher 3-year LFS and OS than patients who received chemotherapy as a bridge to allo-HSCT. Eight patients were not bridged to allo-HSCT after CAR-T infusion, and six (75%) of them remained in remission with a median follow-up of 23.0 months after CAR-T infusion. This observation indicates the effectiveness of CAR-T therapy in improving long-term survival. In this study, the incidence of CRS was 53.8%, which is lower than that reported by Maude et al. (77–93%) (9), suggesting the possible correlation between CRS incidence and severity with tumor burden (24). As present, this study is the first prospective trial to prove the effectiveness of CAR-T therapy in patients with MRD.

The high MRD negativity and survival rates demonstrate that CAR-T therapy is effective in patients with low tumor loads. In patients with MRD, the LFS of patients who were bridged to allo-HSCT was similar to that of patients who were not (n = 8) (75.0% *vs.* 75.0%, P = 0.765). Six (75%) of patients who received CAR-T therapy without bridging to allo-HSCT remained in remission with a median follow-up of 23.0 months after CAR-T infusion. Thus, CAR-T therapy alone with improved CAR-T structure and risk stratification to achieve long-term survival may be feasible in patients with MRD in CR1. The role of allo-HSCT in patients receiving CAR-T therapy for R/R ALL is controversial. In a single-center phase I trial conducted by the University of Pennsylvania, only 10% of MRD-negative patients in CR underwent allo-HSCT post-CAR-T treatment (22). Similarly, in the multicenter ELIANA trial, 14% (n = 8) of the patients in CR underwent allo-HSCT (9); an updated analysis of the trial showed that the OS was nearly identical irrespective of whether patients were censored during allo-HSCT. In an analysis from Seattle Children’s Hospital, the 28% of patients who underwent allo-HSCT after CAR-T therapy had a lower relapse rate than those who did not (18% *vs.* 55%) at a median follow-up of 12.2 months (25). Consistently, an NCI phase I cohort study revealed that most patients (83%) who received CAR-T therapy achieved MRD-negative CR after allo-HSCT, and all HSCT recipients were in remission at the last follow-up (18). Moreover, Hay et al. found that the intervention involving CAR-T therapy with HSCT was associated with improved LFS compared with the non-HSCT intervention (HR 0.39) (26). The American Society for Transplantation and Cellular Therapy recommended that conducting allo-HSCT after CAR-T therapy should be based on patient (physical condition and donor availability), disease (MRD status and B cell aplasia), and CAR T cell (costimulatory domain and potential persistence of CAR T cells) factors (27). Certainly, whether patients with MRD should receive allo-HSCT after CAR-T therapy remains unclear. The present study showed that allo-HSCT might not be necessary in patients with MRD after CAR-T therapy, especially in patients who achieved MRD negativity after CAR-T infusion.

## Conclusions

This prospective study showed that CAR-T therapy could effectively and safely eliminate MRD and significantly improve survival in children with persistent/recurrent MRD in CR1. In some patients, improve survival through CAR-T alone may be possible; however, further multicenter, prospective clinical trials are needed.

## Data Availability Statement

The original contributions presented in the study are included in the article/supplementary material. Further inquiries can be directed to the corresponding authors.

## Ethics Statement

This study was approved by the Peking University People’s Hospital Review Board. All patients’ legal guardians provided written informed consent documents in accordance with the Declaration of Helsinki.

## Author Contributions

LZ and XH designed the research and revised the paper. GH and YFC analyzed the data and wrote the paper. YZ, YJC, PS, JW, YJ, AL, YL, YW (10th author), SJ, LZ, XZ, CY, LX, XZ, KL, and YW (18th author) collected and analyzed data. All authors contributed to the article and approved the submitted version.

## Funding

This work was supported by the Foundation of 2018 Beijing Key Clinical Specialty Construction Project-Pediatrics (2199000726) and the Foundation of CAMS Innovation Fund for Medical Sciences (CIFMS) (grant number: 2019-I2M-5-034).

## Conflict of Interest

Authors YL and YW (10th author) were employed by Beijing Yongtai Reike Biotechnology Company Ltd.

The remaining authors declare that the research was conducted in the absence of any commercial or financial relationships that could be construed as a potential conflict of interest.

## Publisher’s Note

All claims expressed in this article are solely those of the authors and do not necessarily represent those of their affiliated organizations, or those of the publisher, the editors and the reviewers. Any product that may be evaluated in this article, or claim that may be made by its manufacturer, is not guaranteed or endorsed by the publisher.

## References

[1] Malard F Mohty M. Acute Lymphoblastic Leukaemia. Lancet (2020) 395:1146–62. doi: 10.1016/S0140-6736(19)33018-1 32247396

[2] Inaba H Mullighan CG. Pediatric Acute Lymphoblastic Leukemia. Haematologica (2020) 105:2524–39. doi: 10.3324/haematol.2020.247031 PMC7604619 33054110

[3] Berry DA Zhou S Higley H Mukundan L Fu S Reaman GH. Association of Minimal Residual Disease With Clinical Outcome in Pediatric and Adult Acute Lymphoblastic Leukemia: A Meta-Analysis. JAMA Oncol (2017) 3:e170580. doi: 10.1001/jamaoncol.2017.0580 28494052 PMC5824235

[4] Xue YJ Wang Y Jia YP Zuo YX Wu J Lu AD. The Role of Minimal Residual Disease in Specific Subtypes of Pediatric Acute Lymphoblastic Leukemia. Int J Hematol (2021) 113:547–55. doi: 10.1007/s12185-020-03063-w 33386596

[5] Dhedin N Huynh A Maury S Tabrizi R Beldjord K Asnafi V. Role of Allogeneic Stem Cell Transplantation in Adult Patients With Ph-Negative Acute Lymphoblastic Leukemia. Blood (2015) 125:2486–96; quiz 2586. doi: 10.1182/blood-2014-09-599894 25587040

[6] Giebel S Labopin M Socie G Beelen D Browne P Volin L. Improving Results of Allogeneic Hematopoietic Cell Transplantation for Adults With Acute Lymphoblastic Leukemia in First Complete Remission: An Analysis From the Acute Leukemia Working Party of the European Society for Blood and Marrow Transplantation. Haematologica (2017) 102:139–49. doi: 10.3324/haematol.2016.145631 PMC5210244 27686376

[7] Leung W Pui CH Coustan-Smith E Yang J Pei D Gan K. Detectable Minimal Residual Disease Before Hematopoietic Cell Transplantation is Prognostic But Does Not Preclude Cure for Children With Very-High-Risk Leukemia. Blood (2012) 120:468–72. doi: 10.1182/blood-2012-02-409813 PMC3398757 22517895

[8] Inaba H Pui CH. Immunotherapy in Pediatric Acute Lymphoblastic Leukemia. Cancer Metastasis Rev (2019) 38:595–610. doi: 10.1007/s10555-019-09834-0 31811553 PMC6995750

[9] Maude SL Laetsch TW Buechner J Rives S Boyer M Bittencourt H. Tisagenlecleucel in Children and Young Adults With B-Cell Lymphoblastic Leukemia. N Engl J Med (2018) 378:439–48. doi: 10.1056/NEJMoa1709866 PMC5996391 29385370

[10] Chen Y Cheng Y Suo P Yan C Wang Y Chen Y. Donor-Derived CD19-Targeted T Cell Infusion Induces Minimal Residual Disease-Negative Remission in Relapsed B-Cell Acute Lymphoblastic Leukaemia With No Response to Donor Lymphocyte Infusions After Haploidentical Haematopoietic Stem Cell Transplantation. Br J Haematol (2017) 179:598–605. doi: 10.1111/bjh.14923 29076142

[11] Huang R Li X He Y Zhu W Gao L Liu Y. Recent Advances in CAR-T Cell Engineering. J Hematol Oncol (2020) 13:86. doi: 10.1186/s13045-020-00910-5 32616000 PMC7333410

[12] Wang Y Liu QF Xu LP Liu KY Zhang XH Ma X. Haploidentical *vs.*Identical-Sibling Transplant for AML in Remission: A Multicenter, Prospective Study. Blood (2015) 125:3956–62. doi: 10.1182/blood-2015-02-627786 25940714

[13] Huang XJ Liu DH Liu KY Xu LP Chen H Han W. Haploidentical Hematopoietic Stem Cell Transplantation Without *In Vitro* T-Cell Depletion for the Treatment of Hematological Malignancies. Bone Marrow Transplant (2006) 38:291–7. doi: 10.1038/sj.bmt.1705445 16883312

[14] Xue YJ Suo P Huang XJ Lu AD Wang Y Zuo YX. Superior Survival of Unmanipulated Haploidentical Haematopoietic Stem Cell Transplantation Compared With Intensive Chemotherapy as Post-Remission Treatment for Children With Very High-Risk Philadelphia Chromosome Negative B-Cell Acute Lymphoblastic Leukaemia in First Complete Remission. Br J Haematol (2020) 188:757–67. doi: 10.1111/bjh.16226 31725190

[15] Chang YJ Wang Y Xu LP Zhang XH Chen H Chen YH. Haploidentical Donor is Preferred Over Matched Sibling Donor for Pre-Transplantation MRD Positive ALL: A Phase 3 Genetically Randomized Study. J Hematol Oncol (2020) 13:27. doi: 10.1186/s13045-020-00860-y 32228710 PMC7106867

[16] Yang X Wang GX Zhou JF. CAR T Cell Therapy for Hematological Malignancies. Curr Med Sci (2019) 39:874–82. doi: 10.1007/s11596-019-2118-z 31845217

[17] Grupp SA Kalos M Barrett D Aplenc R Porter DL Rheingold SR. Chimeric Antigen Receptor-Modified T Cells for Acute Lymphoid Leukemia. N Engl J Med (2013) 368:1509–18. doi: 10.1056/NEJMoa1215134 PMC4058440 23527958

[18] Lee DW Kochenderfer JN Stetler-Stevenson M Cui YK Delbrook C Feldman SA. T Cells Expressing CD19 Chimeric Antigen Receptors for Acute Lymphoblastic Leukaemia in Children and Young Adults: A Phase 1 Dose-Escalation Trial. Lancet (2015) 385:517–28. doi: 10.1016/S0140-6736(14)61403-3 PMC7065359 25319501

[19] Heng G Jia J Li S Fu G Wang M Qin D. Sustained Therapeutic Efficacy of Humanized Anti-CD19 Chimeric Antigen Receptor T Cells in Relapsed/Refractory Acute Lymphoblastic Leukemia. Clin Cancer Res (2020) 26:1606–15. doi: 10.1158/1078-0432.CCR-19-1339 31732519

[20] Park JH Riviere I Gonen M Wang X Senechal B Curran KJ. Long-Term Follow-Up of CD19 CAR Therapy in Acute Lymphoblastic Leukemia. N Engl J Med (2018) 378:449–59. doi: 10.1056/NEJMoa1709919 PMC6637939 29385376

[21] Cappell KM Sherry RM Yang JC Goff SL Vanasse DA McIntyre L. Long-Term Follow-Up of Anti-CD19 Chimeric Antigen Receptor T-Cell Therapy. J Clin Oncol (2020) 38:3805–15. doi: 10.1200/JCO.20.01467 PMC7655016 33021872

[22] Maude SL Frey N Shaw PA Aplenc R Barrett DM Bunin NJ. Chimeric Antigen Receptor T Cells for Sustained Remissions in Leukemia. N Engl J Med (2014) 371:1507–17. doi: 10.1056/NEJMoa1407222 PMC4267531 25317870

[23] Pan J Yang JF Deng BP Zhao XJ Zhang X Lin YH. High Efficacy and Safety of Low-Dose CD19-Directed CAR-T Cell Therapy in 51 Refractory or Relapsed B Acute Lymphoblastic Leukemia Patients. Leukemia (2017) 31:2587–93. doi: 10.1038/leu.2017.145 28490811

[24] Gust J Hay KA Hanafi LA Li D Myerson D Gonzalez-Cuyar LF. Endothelial Activation and Blood-Brain Barrier Disruption in Neurotoxicity After Adoptive Immunotherapy With CD19 CAR-T Cells. Cancer Discov (2017) 7:1404–19. doi: 10.1158/2159-8290.CD-17-0698 PMC5718945 29025771

[25] Gardner RA Finney O Annesley C Brakke H Summers C Leger K. Intent-To-Treat Leukemia Remission by CD19 CAR T Cells of Defined Formulation and Dose in Children and Young Adults. Blood (2017) 129:3322–31. doi: 10.1182/blood-2017-02-769208 PMC5482103 28408462

[26] Hay KA Gauthier J Hirayama AV Voutsinas JM Wu Q Li D. Factors Associated With Durable EFS in Adult B-Cell ALL Patients Achieving MRD-Negative CR After CD19 CAR T-Cell Therapy. Blood (2019) 133:1652–63. doi: 10.1182/blood-2018-11-883710 PMC6460418 30728140

[27] Kansagra AJ Frey NV Bar M Laetsch TW Carpenter PA Savani BN. Clinical Utilization of Chimeric Antigen Receptor T Cells in B Cell Acute Lymphoblastic Leukemia: An Expert Opinion From the European Society for Blood and Marrow Transplantation and the American Society for Blood and Marrow Transplantation. Biol Blood Marrow Transplant (2019) 25:e76–85. doi: 10.1016/j.bbmt.2018.12.068 PMC8335749 30576834

